# The HtrA protease of *Borrelia burgdorferi* degrades outer membrane protein BmpD and chemotaxis phosphatase CheX

**DOI:** 10.1111/mmi.12213

**Published:** 2013-04-09

**Authors:** James L Coleman, Jameson T Crowley, Alvaro M Toledo, Jorge L Benach

**Affiliations:** 1 New York State Department of Health, Stony Brook UniversityStony Brook, NY, 11794-5120, USA; 2 Department of Molecular Genetics and Microbiology, Center for Infectious Diseases, Stony Brook UniversityStony Brook, NY, 11794-5120, USA

## Abstract

*Borrelia burgdorferi*, the spirochaetal agent of Lyme disease, codes for a single HtrA protein, HtrABb (BB0104) that is homologous to DegP of *Escherichia coli* (41% amino acid identity). HtrABb shows physical and biochemical similarities to DegP in that it has the trimer as its fundamental unit and can degrade casein via its catalytic serine. Recombinant HtrABb exhibits proteolytic activity *in vitro*, while a mutant (HtrABbS198A) does not. However, HtrABb and DegP have some important differences as well. Native HtrABb occurs in both membrane-bound and soluble forms. Despite its homology to DegP, HtrABb could not complement an *E. coli* DegP deletion mutant. Late stage Lyme disease patients, as well as infected mice and rabbits developed a robust antibody response to HtrABb, indicating that it is a B-cell antigen. In co-immunoprecipitation studies, a number of potential binding partners for HtrABb were identified, as well as two specific proteolytic substrates, basic membrane protein D (BmpD/BB0385) and chemotaxis signal transduction phosphatase CheX (BB0671). HtrABb may function in regulating outer membrane lipoproteins and in modulating the chemotactic response of *B. burgdorferi*.

## Introduction

*Borrelia burgdorferi* colonizes many organs that include the skin, the heart, the joints and the nervous system. This systemic tendency requires efficient dissemination to penetrate biological barriers such as the endothelium, the basement membrane surrounding the vasculature and the extracellular matrix, where these organisms reside. Although the striking motility of *B. burgdorferi* is their primary motor for dissemination (Charon *et al*., 2012), most bacteria also use secreted or surface exposed proteases for dissemination, and many secreted proteases are well known virulence factors (for review: Ingmer and Brondsted, 2009).

The genome of *B. burgdorferi* indicates the presence of several proteases that have homologues to those of other bacteria and can have known or inferred physiological functions (Guyard *et al*., 2006; Coleman *et al*., 2009; Kumru *et al*., 2011). However, the Borreliae as a genus have not been shown to produce secreted proteases that assist in dissemination. In contrast, there is a substantial literature from several laboratories that has shown that these organisms use the plasminogen activation system in dissemination (Coleman *et al*., 1997; Gebbia *et al*., 1999; Nordstrand *et al*., 2001; Brissette *et al*., 2009), and in degradation of extracellular matrix (Coleman *et al*., 1999). In fact, the Borreliae not only utilize plasmin but also modulate and induce the production of urokinase plasminogen activator (Coleman *et al*., 2001; Coleman and Benach, 2003; Haile *et al*., 2006; Hovius *et al*., 2009) and its inhibitors (Haile *et al*., 2006). The utilization of the plasminogen activation system by the Borreliae is a prime example of host–pathogen interaction for the establishment of infection.

The HtrA (High Temperature Requirement A) family of serine proteases can be found in all cells from prokaryotes to primates. A unifying feature of this family is the proteolytic domain (Ser-His-Asp catalytic triad) and either one or two C-terminal PDZ domains that mediate protein–protein interactions. The PDZ structural domain consists of about 80–98 amino acids common in signalling proteins, arranged in six β-strands and two α-helices [for reviews: (Pallen and Wren, 1997; Clausen *et al*., 2011)].

DegP, the first HtrA protease characterized from *Escherichia coli* (Swamy *et al*., 1983), is located in the periplasm, where it can function as a chaperone during a protein folding stress response (Ehrmann and Clausen, 2004; Raivio, 2005) and also degrade misfolded proteins. The fundamental structural unit of DegP is a trimer (Krojer *et al*., 2002), which auto-oligomerizes into hexamers that are thought to represent the resting state. Binding of misfolded proteins transforms DegP hexamers into large active, macromolecular structures of 12–24 meric multimers forming a cage that can function as a chaperone protecting the traffic of outer membrane proteins through the periplasm or as a protease (Krojer *et al*., 2008). A recent study has shown that substrate binding can also convert inactive DegP trimers into proteolytically active trimers (Kim and Sauer, 2012). These are critical functions for the HtrA-DegP homologues. Proteases are important for their protective as well as their regulatory roles. As chaperones, some proteases protect other proteins from degradation in the periplasm, and as proteases they can degrade them as well (Sawa *et al*., 2010; Merdanovic *et al*., 2011).

Two areas of interest in our laboratory came together to characterize the single HtrA (BB0104) protease of *B. burgdorferi*. Our long-standing interest in the proteases of *B. burgdorferi* and its use of borrowed proteolysis through plasmin acquisition coincided with our interest in the lipid content of this organism (LaRocca *et al*., 2010). Our first observation was that BB0104, the HtrA homologue (HtrABb), was a component of *B. burgdorgeri* membrane vesicles indicating a possible location within or associated with the outer membrane (Toledo *et al*., 2012), as well as within the periplasm. A role for HtrABb as an outer membrane protease could be very important in helping Borrelia adapt from the vector tick stage into the mammalian stage through a chaperone role for newly produced proteins or by degrading others as protein expression changes during the transition of hosts.

In this study, we show that HtrABb is present in *B. burgdorferi* in membrane-bound and soluble forms. Purified recombinant HtrABb has *in vitro* proteolytic activity that is lacking in the active site Ser→Ala recombinant mutant. Although HtrABb has 41% amino acid identity with the DegP of *E. coli*, it could not complement an *E. coli degP* deletion mutant in the appropriate assays. Possible binding partners for HtrABb were identified by co-immunoprecipitation, and of these partners, basic membrane protein D, BmpD (BB0385) and the chemotaxis signal transduction phosphatase, CheX (BB0671), were shown to be substrates for proteolytic activity.

## Results

**Fig. 1:**
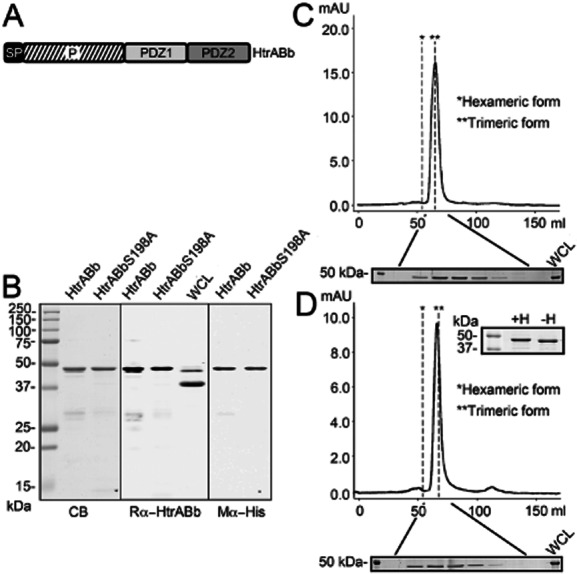
Purified recombinant *B. burgdorferi* HtrA exists predominately as a trimer in the absence of substrate. A. Schematic diagram showing the location of the various functional domains of HtrABb (SP, signal peptide, P, protease domain). B. Recombinant wild-type (HtrABb) and mutant (HtrABbS198A) purified protein was analysed by SDS-PAGE (12.5%) and Western blot. The left panel shows a 0.1% Coomassie blue (CB)-stained gel in which 1 μg of protein was loaded. The middle panel is a Western blot of protein (1 μg) transferred to nitrocellulose from 12.5% SDS-PAGE, showing recognition of recombinant wild-type (HtrABb), recombinant mutant (HtrABbS198A) and native whole-cell lysate (WCL)-derived HtrABb by rabbit anti-HtrABb polyclonal antibody (Rα-HtrABb). The Rα-HtrABb cross-reacted with FlaB in the WCL (lower band). The right panel is a Western blot showing recognition of recombinant wild-type and mutant HtrABb by mouse anti-His tag antibody (Mα-His). Lanes receiving recombinant HtrAs, received 0.15 μg of protein, while the WCL lane (*B. burgdorferi* strain B31A3) received 15 μg. Secondary antibodies were IRDye goat anti-rabbit IgG 700DX and IRDye goat anti-mouse IgG 800CW (Rockland Immunochemicals, Gilbertville, PA). C. The superdex-200 size exclusion chromatography (SEC) elution profile of recombinant HtrABb containing intact N-terminal His-tag is shown. SDS-PAGE verified that HtrA is present in the collected fractions. The expected elution volumes for the hexameric form (56.6 ml) and trimeric form (64.8 ml) are shown by the dashed lines. D. SEC profile of *B. burgdorferi* without N-terminal His-tag is shown. SDS-PAGE verified that HtrA is present in the collected fractions. The expected elution volumes were 57.1 ml for the hexameric form and 65.4 ml for the trimeric form. Inset, Coomassie blue stained SDS-PAGE showing wild-type HtrABb before (+ H) and after (− H) His-tag removal. The results shown in (C) and (D) are each representative of two independent experiments.

HtrABb, the DegP homologue in *B. burgdorferi*, is a chromosomally encoded polypeptide with a predicted molecular mass of approximately 52 kDa upon synthesis. The sequence contains a predicted signal peptidase I cleavage site between residues Ala^28^ and Ser^29^ (Petersen *et al*., 2011) (probability: 0.988, Hidden Markov Model, Signal 3.0 Server) and a chymotrypsin-like proteolytic domain containing a putative catalytic serine (S^198^). In addition, two characteristic PDZ domains are predicted to exist near the HtrABb C-terminus (Prosite: http://prosite.expasy.org) (Fig. 1A). Although PDZ domains mainly provide for protein–protein interactions, there is evidence that they can also interact with phosphoinositide signalling lipids in cell membranes (Zimmermann *et al*., 2002).

### Recombinant HtrABb assumes oligomeric forms characteristic of a trimer

As an initial step in addressing the function(s) of HtrABb and its contribution to Lyme disease pathogenesis, we sought to generate an HtrA-null mutant for use in mouse infection studies. Despite repeated attempts we were unable to create a mutant, thus necessitating the use of alternative approaches.

Therefore, to characterize HtrABb and assess its biological activity we over expressed HtrABb in *E. coli* and obtained purified soluble recombinant protein (Fig. 1B, left panel) (PCR primers, plasmids and *E. coli* strains are given in Tables S1 and S2 respectively). To obtain full expression of the recombinant protein, facilitate its solubility and to prevent its mislocalization, only the DNA coding for the predicted mature protein (minus the leader peptide) was cloned into the expression vector. By use of site-directed mutagenesis, the putative catalytic serine (S^198^) was mutated to alanine to abolish proteolytic activity. Purified mutant recombinant protein (HtrABbS198A) was generated as it was for the wild-type (Fig. 1B, left panel). Rabbit antiserum raised against the wild-type recombinant HtrA protein recognized both the wild-type and mutant HtrA, as well as the HtrA from *B. burgdorferi* whole-cell lysate (Fig. 1B, middle panel). The rabbit antiserum cross-reacted with an antigen that also bound a monoclonal antibody specific for FlaB (p41) in the whole-cell lysate (Fig. 1B, middle panel, lower band). The reactivity of monoclonal antibody specific for the 6× His-tag is shown in Fig. 1B, right panel.

Following synthesis, *E. coli* DegP molecules auto-assemble into trimers, the protein's fundamental structural unit. The binding of substrate is required to induce further aggregation into still larger, proteolytically competent forms (Singh *et al*., 2011). To establish the oligomeric state of HtrABb, recombinant protein (with His-tag intact) was fractionated by size exclusion chromatography. The expected elution volumes for HtrABb (with His-Tag intact) were calculated to be 56.6 ml for the hexameric form and 64.8 ml for the trimeric form. HtrABb eluted from the column in a single peak, at a volume of 65.4 ml, indicative of a trimer. Peak fractions were collected and analysed by SDS-PAGE, which verified that HtrABb was present in the collected fractions (Fig. 1C). To verify that the N-terminal His-tag was not interfering with the protein achieving its native oligomeric state, the His-tag was cleaved off using thrombin (Fig. 1D, inset) and the resultant cleaved protein was also analysed by size exclusion chromatography. The expected elution volumes were calculated to be 57.1 ml for the hexamer and 65.4 ml for the trimer. Cleaved HtrABb eluted at a volume of 66.6 ml (Fig. 1D). Thus, the elution profile for the cleaved HtrABb was not different from the trimeric His-tagged form.

### Recombinant HtrABb is an active protease

**Fig. 2:**
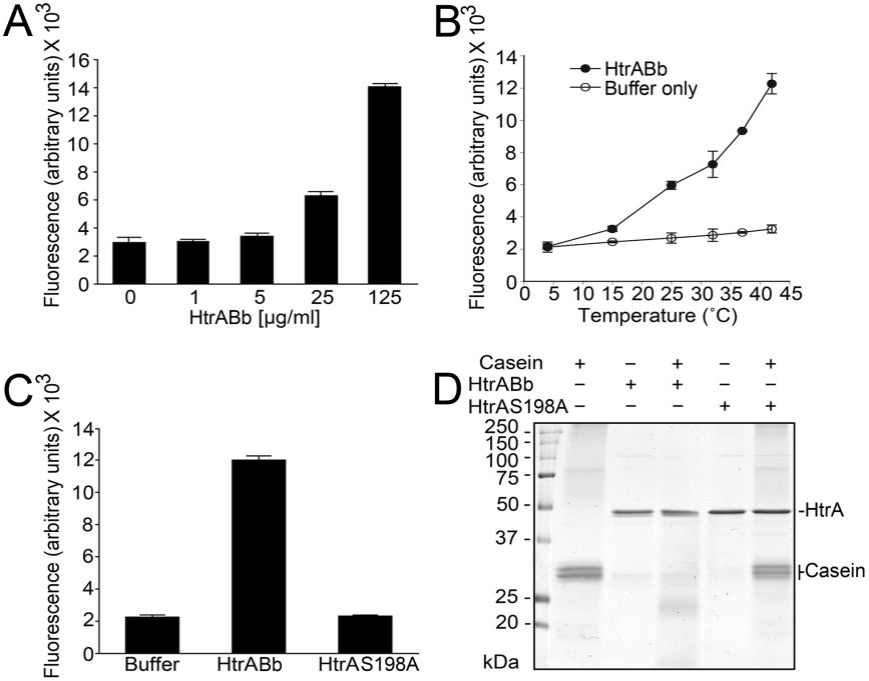
Recombinant HtrABb is catalytically active. A. Degradation of FITC-labelled casein by HtrABb is concentration-dependent. B. Degradation of FITC-labelled casein by HtrABb is temperature-dependent. C. Mutant (HtrABbS198A) (125 μg ml^−1^) does not degrade FITC-labelled casein. Error bars represent the mean ± standard deviation of triplicate proteolytic digestions of representative experiments. D. SDS-PAGE (12.5%) analysis of HtrABb and HtrABbS198A degradation of casein is shown. The gel was stained with 0.1% Coomassie blue.

To investigate the catalytic potential of recombinant HtrABb and HtrABbS198A, we conducted caseinolytic assays using FITC-labelled casein. The use of casein to assess for HtrA enzymatic activity is advantageous in that it contains a high proportion of proline residues, which do not interact with each other. In addition, there are also no disulphide bridges. Consequently, there is little or no tertiary structure and the molecule therefore mimics an unfolded substrate. HtrABb-mediated degradation of casein-FITC occurred in a concentration- and temperature-dependent manner (Fig. 2A and B). In contrast, the mutant protein HtrABbS198A did not show activity at levels above that of the buffer control (Fig. 2C). The results shown in panels A–C were validated in separate experiments using unconjugated casein followed by SDS-PAGE (Fig. 2D). The caseinolytic assays confirmed the catalytic activity predicted for HtrABb by its amino acid sequence. Moreover, through targeted mutagenesis of HtrABb S^198^, the catalytic serine residue was shown to be identified correctly.

### HtrABb is a component of *B. burgdorferi* vesicles

**Fig. 3:**
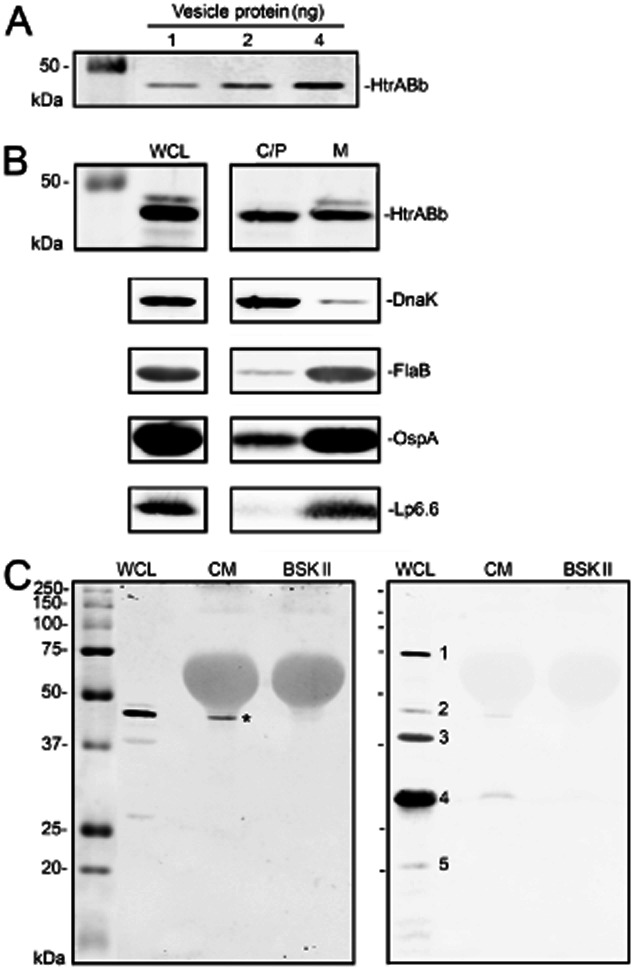
HtrABb is a component of *B. burgdorferi* vesicles and is also present in soluble form. A. Increasing amounts of purified *B. burgdorferi* vesicle protein was separated by 12.5% SDS-PAGE and transferred to nitrocellulose. HtrABb was detected by Western blot using rabbit anti-HtrABb polyclonal antiserum. B. Total membrane and periplasm/cytoplasm fractions were prepared from *B. burgdorferi*. Fractions (derived from approximately 4 × 10^7^ spirochaetes) and *B. burgdorferi* whole cell lysate were separated by 12.5% SDS-PAGE transferred to nitrocellulose. The membrane was probed initially with rabbit anti-HtrABb (upper 2 panels) and re-probed with monoclonal antibodies to *B. burgdorferi* DnaK, FlaB, OspA and LP6.6 (lower panels). WCL, whole-cell lysate; C/P, cytoplasm/periplasm; M, total membranes. C. Soluble HtrABb is present in *B. burgdorferi* conditioned medium. Cell/ vesicles-free conditioned medium (2 μl) obtained from *B. burgdorferi* cultures was separated by 12.5% SDS-PAGE and transferred to nitrocellulose. Left panel, the nitrocellulose was probed with rabbit anti-HtrABb. Asterisk, HtrABb. Right panel, the membrane was re-probed with monoclonal antibodies to DnaK (1), FlaB (3) and OspA (4) and polyclonal antibody to OspC (5). Band 2 is HtraBb. Secondary antibodies were IRDye goat anti-rabbit IgG 700DX and IRDye goat anti-mouse IgG 800CW (Rockland Immunochemicals). WCL, *B. burgdorferi* B31A3 whole-cell lysate; CM, conditioned medium; BSK II, uninoculated BSK II medium. The results shown here are representative of two independent experiments.

In a previous report we found that HtrABb was one of a group of proteins detected by mass spectrometry in isolated *B. burgdorferi* vesicles (Toledo *et al*., 2012). To verify the presence of HtrA in vesicles, these structures were prepared from *B. burgdorferi* cells as previously described (Toledo *et al*., 2012) and tested for the presence of HtrABb. SDS-PAGE and Western blot analysis of vesicles using rabbit polyclonal antibody generated against the recombinant protein demonstrated that HtrA was detectable in as little as 1 ng of vesicle protein (Fig. 3A). However, digestion of vesicles by proteinase K revealed that HtrA is not exposed on the vesicle surface (Toledo *et al*., 2012).

### HtrABb exists in both membrane-bound and soluble forms, and is detectable in conditioned medium

The presence of HtrABb in vesicles led to the question of its distribution between membrane-bound and periplasmic (soluble) forms. To address this question, we separated *B. burgdorferi* cells and their contents into total membrane and total cytoplasm/periplasm fractions by a sonication and ultracentrifugation technique. The individual fractions were analysed by SDS-PAGE and the protein, transferred to nitrocellulose, was tested for the presence of HtrABb by Western blot analysis. HtrA was detected in approximately equal amounts in both the membrane (M) and cytoplasm/periplasm (C/P) fractions (Fig. 3B). The membrane was re-probed with monoclonal antibodies for DnaK (cytoplasm), FlaB (periplasm), and OspA and lp6.6 (membrane). DnaK partitioned almost entirely into the soluble (cytoplasm/periplasm) fraction and FlaB remained associated with the membrane fraction, as did OspA and lp6.6 (Fig. 3B). These results indicated that HtrABb occurs in both soluble and membrane-bound forms. A similar location has been reported for *Helicobacter pylori*, with HtrA being identified in both structure-bound and soluble fractions (Backert *et al*., 2005). Membrane-bound DegP has also been reported in *Bordetella pertussis* (Baud *et al*., 2011), and, in addition to *H. pylori* (Backert *et al*., 2005; Hoy *et al*., 2010), *Bacillus anthracis* (Sela-Abramovich *et al*., 2009) has been shown to produce soluble HtrA. To determine if HtrABb is released extracellularly, mid-log phase *B. burgdorferi* were centrifuged and resuspended in fresh BSK II medium, then incubated for two hr at 33°C. After filtration and ultracentrifugation, the conditioned medium was analysed by SDS-PAGE and Western blot. As a control, an equivalent volume of uninoculated BSK II from the same batch of medium was also processed as described above. B31 whole-cell lysate was used to mark the position of native HtrABb. After probing with rabbit antiserum, HtrA was detected in as little as 2 μl of the conditioned medium, whereas none was detected in the control lane. (Fig. 3C). The membrane was further probed with monoclonal antibodies to DnaK, FlaB, OspA and OspC, all of which were substantially absent in the conditioned medium (Fig. 3C).

### HtrABb is immunogenic across species

**Fig. 4:**
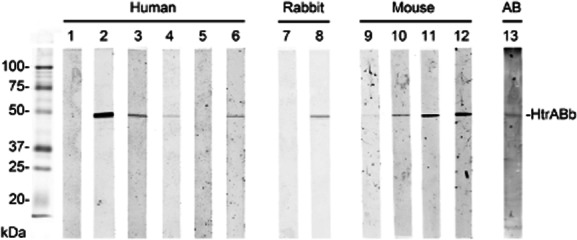
HtrABb is antigenic across species. Purified recombinant HtrABb was transferred from 12.5% SDS-PAGE gels to nitrocellulose. Protein immobilized on nitrocellulose strips was tested by Western blot against Lyme disease patient sera, tick-infected rabbit sera, or needle-infected mouse sera. Lanes: 1, pooled human negative control serum; 2–6, Lyme disease patient sera; 7, rabbit uninfected negative control serum; 8, Tick-infected rabbit serum; 9, mouse uninfected negative control serum; 10–12, needle-infected mouse sera; 13, amido black stain for total protein. All sera were tested at a dilution of 1:100. Secondary antibodies were either IRDye goat anti-human IgG 800, IRDye goat anti-rabbit IgG 700DX or IRDye goat anti-mouse IgG 800CW (Rockland Immunochemicals).

HtrA is highly immunogenic in *Haemophilus influenzae* and has been proposed as a vaccine candidate (Loosmore *et al*., 1998). Additionally, the presence of HtrABb in *B. burgdorferi* vesicles could be of significance in that vesicles are known to release their cargo proteins, thus, making it accessible for recognition by the immune response. To determine if HtrABb is an immunogen, we electrophoresed recombinant HtrABb and transferred the protein to nitrocellulose. Sera from patients with Lyme arthritis (n = 5) were tested by Western blot, and four out of the five-showed reactivity to HtrABb. Pooled negative control serum did not recognize HtrABb (Fig. 4). Serum from an *Ixodes scapularis* tick-infected rabbit and needle-infected mice also recognized the recombinant HtrABb while negative control sera did not (Fig. 4). These results indicate that HtrABb can elicit an antibody response during Borrelia infection and, thus may play a role in the immunopathogenesis of Lyme disease.

### HtrABb does not complement an *E. coli* DegP mutant

**Fig. 5:**
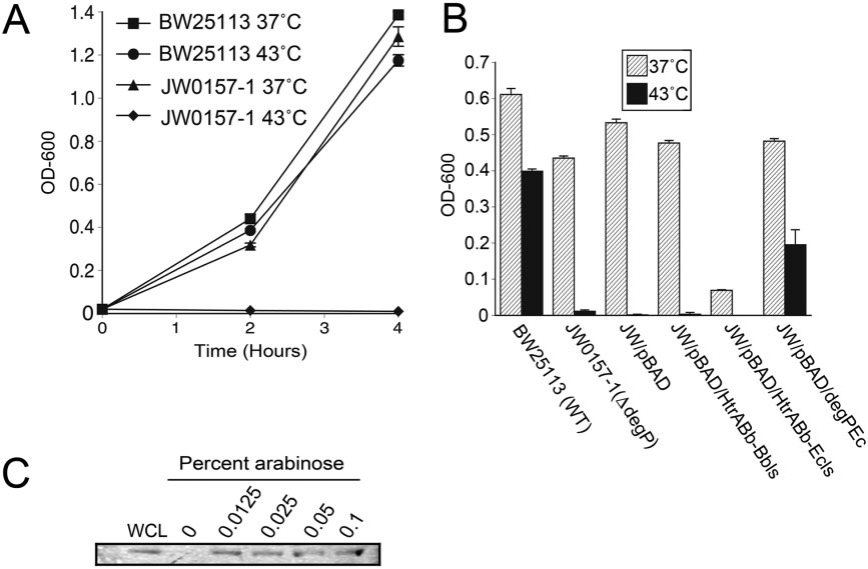
HtrABb does not complement DegP in an *E. coli* *degP* mutant. A. The growth of *E. coli* parental strain BW25113 and Δ*degP* mutant strain JW0157-1 in LB medium at 37°C and 43°C is shown. B. The growth of strain BW25113, strain JW0157-1, JW0157-1 complemented with empty vector alone (JWpBAD), JW0157-1 complemented with HtrABb and *B. burgdorferi* leader sequence (JW/pBAD/htrABb-Bbls), JW0157-1 complemented with HtrABb and *E. coli* leader sequence (JW/pBAD/htrABb-Ecls) and JW0157-1 complemented with *E. coli* degP (JW/pBAD/degPEc) at 37°C and 43°C is shown. With the exception of BW25113 and JW0157-1, all *E. coli* were grown in the presence of 0.2% arabinose. Error bars represent the mean ± standard deviation of combined data from three separate experiments. C. SDS-PAGE (15%) and Western blot showing expression of wild-type HtrABb in *E. coli* JW/pBAD/HtrABb-Ecls cultured at 37°C in LB containing a range of arabinose concentrations. WCL, B31A3 whole-cell lysate. *E. coli* were grown at 37°C for 5 h in LB medium with indicated concentrations of arabinose, washed 1× in DPBS and resuspended in 50 μl of 1× SDS-PAGE sample buffer. Fifteen microlitres of each sample was loaded. The primary antibody was rabbit anti-HtrABb. The secondary antibody was IRDye goat anti-rabbit IgG 700DX.

HtrABb showed the highest identity with the DegP of *E. coli* (41%, Table 1). To gain insights into the potential role(s) of HtrABb in *B. burgdorferi*, we used a complementation strategy to determine if HtrABb was functionally analogous to DegP in *E. coli*. The *htrABb* ORF was cloned into the multiple cloning site of expression plasmid pBAD24 (Guzman *et al*., 1995) and included the DNA coding for the Borrelia HtrA leader sequence or the *E. coli degP* leader sequence to form plasmids pBAD/*htrABb-*Bbls and pBAD/*htrABb*-Ecls respectively. Both pBAD/*htrABb-*Bbls and pBAD/*htrA*Bb-Ecls were transformed into DegP-deficient strain JW0157-1, in which *degP* is deleted by a *kan*-cassette, to form strains JW/pBAD/HtrABb-Bbls and JW/pBAD/HtrABb-Ecls. JW0157-1 grows well at 37°C but does not grow at 43°C, while wild-type strain BW25113 grows well at both temperatures, thus providing for a clear and reliable phenotype (Lipinska *et al*., 1989, Seol *et al*., 1991) (Fig. 5A). In growth experiments conducted at 37°C and 43°C, neither construct was able to complement JW0157-1 at 43°C. However, the two strains behaved differently in culture; JW/pBAD/HtrABb-Bbls grew well at 37°C but did not grow at 43°C. This strain also expressed little or no HtrABb protein at 37°C as measured by Western blot (data not shown). This is likely due to an inability by *E. coli* to recognize the *B. burgdorferi* HtrA leader sequence, resulting in mislocalization and/or degradation of the protein. Strain JW/pBAD/HtrABb-Ecls grew poorly at 37°C and not at all at 43°C (Fig. 5B). Analysis of this strain by SDS-PAGE after growth at 37°C showed that it expressed full length HtrABb at a range of arabinose concentrations (Fig. 5C). The expression of HtrA at 37°C and the failure to grow to a high density at the same temperature by JW/pBAD/HtrABb-Ecls suggests that the HtrABb is toxic to the *E. coli* cell. Experiments using lower concentrations of arabinose showed the same lack of complementation (data not shown). Lastly, complementation of JW0157-1 with *E. coli degP* (plasmid pBAD/*degPEc*) to form strain JW/pBAD/degPEc resulted in a 42% recovery of the 37°C growth phenotype and therefore validated the complementation strategy (Figs 5B and S1).

**Table 1:** Identities of amino acid sequences of *E. coli* Deg proteases and *H. pylori*, *B. subtilis*, *T. pallidum*, *L. interrogans*, *B. burgdorferi* and *B. hermsii* HtrA proteases

	% Identity^a^													
	
	1^b^	2	3	4	5	6	7	8	9	10	11	12	13	14
1. *E. coli* DegS	100													
2. *E. coli* DegP	39.5	100												
3. *E. coli* DegQ	41.2	67.8	100											
4. *H. pylori* HtrA	41.2	46	42.8	100										
5. *B. subtilis* HtrA	32.2	33.4	33.4	37.3	100									
6. *B. subtilis* HtrB	31.2	35.7	34.7	37.9	46.3	100								
7. *B. subtilis* HtrC	34.4	37	34.1	37.9	47.6	60.8	100							
8. *T. pallidum* HtrA2	28.3	38.9	36.7	32.5	34.4	33.4	35	100						
9. *T. pallidum* HtrA1	34.4	36.3	36.3	36	35.4	33.4	33.4	33.4	100					
10. *B. burgdorferi* HtrA	25.7	40.8	39.9	33.8	31.2	36.7	36.4	44.4	31.8	100				
11. *L. interrogans* DegQ	33.8	36	35.7	34.7	33.1	32.8	34.4	34.1	35	34.4	100			
12. *L. interrogans* HtrA	28.6	36	34.1	32.5	29.9	31.5	31.2	32.5	28.6	33.8	36.3	100		
13. *B. hermsii* DO^c^	26.4	37.6	38.3	31.5	31.8	34.1	35.4	43.7	32.2	69.1	33.4	30.9	100	
14. *B. hermsii* HhoB^c^	25.3	28.6	27.6	24.7	23.7	25.0	26.3	25.7	23.0	30.3	25.0	26.6	25.3	100

a Identities were calculated from the distance matrix (P-distance values) in a pairwise deletion procedure.

b Horizontal numbers 1–14 match the vertical numbers corresponding to bacterial species.

c Based on sequence homology, *B. hermsii* DO is the homologue of *B. burgdorferi htrA*. *B. hermsii hhoB* (formerly known as *bhpA*) is an orthologue of *B. burgdorferi htrA* and a paralogue of *B. hermsii* DO.

### Basic membrane protein D (BmpD) and chemotaxis phosphatase CheX co-immunoprecipitate with and are substrates for HtrABb

**Fig. 6:**
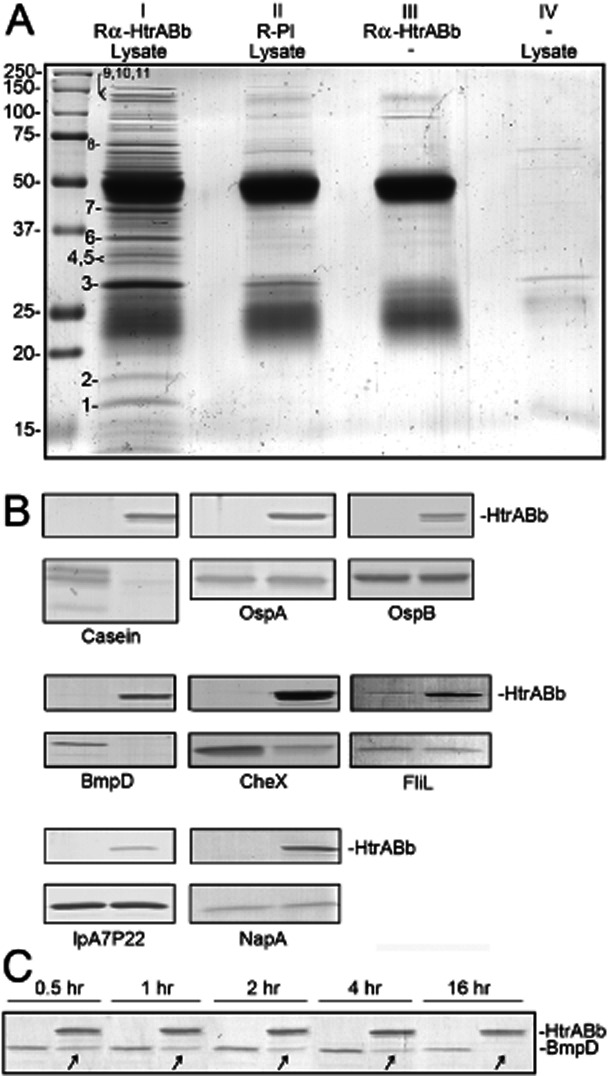
BmpD and CheX are substrates for HtrABb. A. SDS-PAGE (12.5%) analysis of co-immunoprecipitated proteins. Lane I, magnetic Dynabeads coupled with rabbit anti-HtrABb IgG were used to co-immunoprecipitate HtrA along with potential binding partners from *B. burgdorferi* B31A3 lysate. Bands indicated by numbers were cut out of the gel and analysed by LC- mass spectrometry. Bands 4 and 5 were cut out as one, as were bands 9–11. Lane II, pre-immune (PI) serum IgG from the same rabbit was substituted for R-anti-HtrABb IgG. Lane III, the *B. burgdorferi* lysate was omitted (anti-HtrABb IgG antibody-coated Dynabeads were incubated with lysis buffer alone); Lane IV, IgG was omitted (uncoated Dynabeads were incubated with IgG purification kit elution buffer alone). The samples were separated by SDS-PAGE and protein was stained with 0.1% Coomassie blue in 50% methanol, 10% acetic acid. Rα-HtrABb, serum IgG from rabbit immunized with recombinant HtrABb; R-PI, rabbit pre-immune serum IgG. B. *B. burgdorferi* BmpD and CheX are substrates for HtrABb. A number of potential HtrABb binding partners/substrates identified by LC-MS were chosen for further analysis as target proteins for degradation. Proteins (1–4 μg) were digested for 16 h at 37°C with or without 1 μg each of recombinant HtrABb. Digests were separated by SDS-PAGE and the gel was stained with Coomassie blue. For each protein, the presence or absence of the HtrA band is shown in the upper box. Target proteins are lower boxes. C. Time-course of digestion of BmpD by HtrABb. Arrows indicate digested BmpD.

To further investigate HtrABb function, we utilized a co-immunoprecipitation strategy to identify potential HtrABb binding partners and proteolytic targets. Incubation of rabbit anti-HtrABb-bound magnetic beads with *B. burgdorferi* lysate resulted in the identification of a number of protein bands of interest (Fig. 6A, lane I). Co-immunoprecipitated bands present in lane I and absent in the control lanes II-IV were analysed by liquid chromatography/mass spectrometry (Table 2). From this data, OspA, OspB, basic membrane protein (BmpD, BB0385), chemotaxis protein CheX (BB0671), flagellar basal body-associated protein FliL (BB0279), outer surface 22 kDa lipoprotein (antigen lpA7, BB0365) and NapA (BB0690) were chosen to be tested as substrates of HtrABb. HtrABb effectively degraded both BmpD and CheX (Fig. 6B). Digestion of BmpD with HtrABb was time-dependent, and largely complete at 4 h (Fig. 6C). With the exception of the positive control casein, HtrABb exhibited no enzymatic activity toward any of the other proteins tested (Fig. 6B). In separate experiments, the buffers for each of the target proteins, when included in the digestion mixtures, did not interfere with the proteolytic activity of HtrABb against casein (not shown). Since HtrA acts mainly on unfolded or misfolded proteins, we considered the possibility that disrupting the tertiary structure of a protein by denaturing could alter the degradative specificity of HtrABb. To accomplish this, OspA and OspB were selected from the group of HtrABb-resistant proteins, denatured by heating to 56°C or by boiling, and incubated with HtrABb as described above. Neither of these treatments resulted in increased degradation by HtrABb (not shown).

**Table 2:** HtrA-binding *B. burgdorferi* proteins identified by co-immunoprecipitation and liquid chromatography/mass spectrometry

Band^a^	Protein	Gene ID	MW
1	Outer surface protein A (OspA)	BBA15	27 714
1	Outer surface protein B (OspB)	BBA16	30 221
2	Outer surface 22 kDa lipoprotein	BB0365	19 333
2	Neutrophil activating protein (NapA)	BB0690	20 982
2	Flagellar basal body-associated protein (FliL)	BB0279	20 060
2	Chemotaxis protein (CheX)	BB0671	17 614
3	Outer surface protein A (OspA)	BBA15	29 365
6	Basic membrane protein D (BmpD)	BB0385	35 173

a Band numbers correspond to those shown in Fig. 6A.

## Discussion

HtrA family proteins are key players in protein quality control in both eukaryotes and prokaryotes. In prokaryotes they function in the periplasm to degrade or remodel damaged or improperly folded membrane proteins in a tightly controlled manner, and thus increase cellular viability (Spiess *et al*., 1999; Iwanczyk *et al*., 2007; Meltzer *et al*., 2008). The most widely studied members of the prokaryotic HtrA family are DegP (to which *B. burgdorferi* HtrA is most closely related, Table 2), DegS, and DegQ in *E. coli*. These proteins, and all HtrA family proteins, have in common a modular structural organization consisting of an N-terminal chymotrypsin-like proteolytic domain, and a single (DegS) or a pair (DegP, DegQ) of C-terminal PDZ domains in tandem, which, in bacteria, mediate protein–protein interactions through binding to C-termini of target proteins. DegP and DegQ are synthesized initially with N-terminal signal peptides. After transport from the cytosol across the inner membrane via the Sec translocation pathway, they are released into the periplasm upon signal peptide cleavage, where they function in their characteristic roles (Lipinska *et al*., 1990; Waller and Sauer, 1996; Dalbey *et al*., 2012).

HtrABb first drew our interest when it was found to be a constituent of *B. burgdorferi* vesicles (Toledo *et al*., 2012). Many Gram-negative bacteria release vesicles, which contain both outer membrane and periplasmic elements, as part of the bacterial stress response (McBroom and Kuehn, 2007). Thus, the fact that HtrABb was detected within the *B. burgdorferi* vesicles is not surprising because HtrA homologues degrade aberrant proteins that accumulate under stress conditions. For example, in the case of *B. burgdorferi*, vesicles are released in response to the binding of a bactericidal monoclonal antibody to OspB (LaRocca *et al*., 2009).

In addition to vesicles, HtrABb was also detected in conditioned medium after filtration and ultracentrifugation, suggesting its release into the extracellular milieu. This phenomenon is of interest since active export of proteins is not predicted for *B. burgdorferi*, as it does not possess the necessary genes for known secretion systems (Fraser *et al*., 1997). Whether this release occurs *in vivo* is not known, but if it were to be released as a functional protease (regardless of how it occurs) it could be an important virulence mechanism. To support this view, there is evidence for secreted HtrA in other bacteria. HtrA from *H. pylori* is secreted extracellularly and independent of its Type IV secretion system (Tomb *et al*., 1997; Lower *et al*., 2008), and cleaves E-cadherin to disrupt epithelial barriers (Hoy *et al*., 2010). Other pathogens such as *E. coli*, *Campylobacter jejuni* and *Shigella flexneri* also exhibit HtrA-mediated degradation of E-cadherin (Hoy *et al*., 2012).

Our studies show that HtrABb demonstrates physical and biochemical similarities to *E. coli* DegP, reflected by the presence of two PDZ domains, formation of a trimeric fundamental structural unit, and hydrolysis of casein. Although HtrA is active at a wide range of temperatures, as its name indicates (High Temperature Requirement), it works best at the higher end of the range – interestingly, since HtrABb does have proteolytic activity at 20°C, a trait also shared by the *E. coli* homologue (Skorko-Glonek *et al*., 2008), it is possible that it could be active in the unfed tick.

However, HtrABb and DegP have some important differences as well. Notably, the amino acid sequence of *E. coli* DegP contains two cysteine residues, at positions 57 and 69, providing for a disulphide bridge, which is important for maintenance of DegP cohesion (Skorko-Glonek *et al*., 2003), and proteolytic activity (Skorko-Glonek *et al*., 2008). The amino acid sequence of HtrABb contains no cysteine residues, and thus no disulphide bridges, which may reflect differences from *E. coli* in inherent molecular stability. Additionally, where *E. coli* DegP is periplasmic, native HtrABb exists in both membrane-bound and soluble forms. Finally, in a previous study on *B. burgdorferi* Lon1 protease (Coleman *et al*., 2009), we utilized a complementation approach to demonstrate functional similarity with *E. coli* Lon in a Lon-deficient *E. col*i mutant. Using the same strategy, provision of HtrABb did not complement an *E. coli* DegP deletion mutant.

HtrA is a virulence factor in a variety of Gram-negative bacteria, such as *Salmonella enterica* (Baumler *et al*., 1994), *Brucella abortus* (Elzer *et al*., 1996), *Yersinia enterocolitica* (Li *et al*., 1996) and *Streptococcus pyogenes* (Jones *et al*., 2001). We could not address directly the question of whether HtrABb plays a role in *B. burgdorferi* infection because, despite repeated attempts, we were unable to create an HtrA-null mutant, a phenomenon not unique to *B. burgdorferi*, as it has been reported in *H. pylori* as well (Hoy *et al*., 2012). Interestingly, the genomes of both *B. burgdorferi* and *H. pylori* code for only one HtrA homologue, whereas many other bacteria, including the pathogenic spirochaetes *Treponema pallidum*, *Leptospira interrogans* and *Borrelia hermsii*, have two and up to three HtrA homologues (Table 1). Furthermore, HtrA deletions are only known in bacteria that have more than one homologue. The presence in *B. burgdorferi* of a single HtrA homologue would lead to an inevitable lack of functional overlap. Thus, deletion of *htrA* in *B. burgdorferi* may represent a lethal mutation.

The absence of an HtrA knockout led us to pursue other approaches to assess its cellular function and role in Lyme disease pathogenesis. In Western blots, HtrABb was recognized by serum antibody from Lyme disease patients as well as needle-infected mice and an *I. scapularis* tick-infected rabbit, thus identifying it as a B-cell immunogen.

By virtue of its presumed role in cellular maintenance and quality control, HtrABb is likely to interact directly with a number of autologous outer membrane proteins, which may, in turn, influence the course of an infection. In the absence of an isogenic mutant, we sought to identify potential binding partners by the use of co-immunoprecipitation. In independent experiments, at least seven *B. burgdorferi* proteins were consistently identified as HtrABb ligands (Table 2). When these proteins were tested as HtrABb substrates, only basic membrane protein, BmpD (BB0385) and chemotaxis phosphatase, CheX (BB0671) were degraded. HtrA directs its proteolytic activity against misfolded or denatured proteins. In our experiments, we used recombinant proteins as substrates, which could account for the slow rate of proteolysis. The proteolytic effects of HtrA are also influenced by temperature, and by the presence of other unfolded proteins (Cassone *et al*., 2012), so it is possible that there may be more suitable conditions. Importantly, however, differential cleavage of potential substrates shows that HtrABb has the ability to discriminate between proteins and may act as a protease for some and stabilize others by acting as a chaperone. While we did not demonstrate chaperone activity by HtrABb in this study, the known chaperone functions of DegP are in accord with our findings where putative binding partners outnumber the proteolytic substrates (Meltzer *et al*., 2009).

BmpD (BB0385) is closely related to BmpA (BB0383), BmpB (BB0382) and BmpC (BB0384) and to the TmpC of *T. pallidum*, and is present in all species of the *B. burgdorferi* sensu lato complex (Ramamoorthy *et al*., 1996). Transcription and protein expression of *bmp*D were increased during early stationary phase of growth in culture suggesting a specific role at that nutritionally stressful phase (Ramamoorthy and Philipp, 1998). *Bmp*D has its own transcriptional start sites at −74 and −76, and is transcribed in a polycistronic message with ribosomal protein genes, *rps*L-*rps*G and is not co-transcribed with *bmp*A-C (Dobrikova *et al*., 2001). BmpD has important roles in infection as it is expressed in patients and is immunogenic (Bryksin *et al*., 2005). BmpD is also a *B. burgdorferi* adhesin for endothelium and laminin (Antonara *et al*., 2007; Verma *et al*., 2009). This is consistent with a surface location for this lipoprotein. An HtrA-like protease of *Mycobacterium tuberculosis* has been shown to degrade an antigenic outer envelope protein that is involved in the stress response in this organism (White *et al*., 2011). Given its differential expression under stress conditions (Ramamoorthy and Philipp, 1998), we can suggest that proteolysis of BmpD by HtrABb is part of the regulatory mechanism of the stress response in *B. burgdorferi*, which may reflect a similar function with respect to the increasing number of outer membrane lipoproteins with known functions (Kenedy *et al*., 2012)*.*

In most chemotaxis two-component systems, a sensor histidine kinase auto-phosphorylates its histidine residue. The phosphate group is transferred to an aspartyl residue of a response regulator, which becomes an activated DNA-binding protein that controls the sense of flagella rotation and thereby controls swimming behaviour. Deactivation of the response regulator occurs by hydrolysis of the phosphoryl group by a phosphatase. Within this generalized chemotaxis two-component signal transduction system, there is some variability, particularly in the signal termination step marked by the removal of the phosphoryl group from the response regulator by the phophatases. For example, *E. coli* has CheZ as the phosphatase of the response regulator CheY, but *Bacilus subtilis* uses phosphatases of the CheC-FliY-CheX family (Muff *et al*., 2007). *B. burgdorferi* does not have the CheZ prototype phosphatase of *E. coli*. Instead, CheX dephosphorylates the response regulator (CheY). Inactivation of CheX resulted in a spirochaete that flexes continuously but cannot swim or reverse its motion. CheX is a homodimer and is the only phosphatase in the signal transduction pathway of *B. burgdorferi* chemotaxis (Motaleb *et al*., 2005;2011a). Interestingly, the CheX of *B. burgdorferi* is unique in that it has a different mode of binding its response regulator CheY3 (Motaleb *et al*., 2011a) from most other phosphatases of two-component systems (Pazy *et al*., 2010). In addition to the chemotaxis system, *B. burgdorferi* has another two-component system that regulates survival in ticks, and HtrABb could also play a role in this pathway (Sultan *et al*., 2010;2011; Caimano *et al*., 2011).

The degradation of CheX by HtrABb could provide another level of regulation of the chemotaxis two-component system of *B. burgdorferi*. This degradation of CheX could signal the maintenance of the chemotactic response through removal of the phosphatase, and would represent a new substrate for this protease. However, the location of the effectors of the chemotaxis two-component system within bacteria are generally thought to be in the cell membrane (receptor and response regulator), and in the cytosol (response regulator and phosphatase). There is predictive evidence that the CheX of *B. burgdorferi* may straddle several locations in the cell. Four separate predictive algorithms (Table S3) agree on a transmembrane α-helix within amino acid residues 43–60. Furthermore, an external loop (periplasmic) is also predicted for CheX (Table S3). Of note is that most trypsin degradation sites for CheX are in the external loop (amino acid residues 61–161). CheX does not have a predicted signal peptidase cleavage site. The catalytic amino acids E96 and N99 are also in the predicted external loop (Pazy *et al*., 2010). Moreover, CheZ, the CheX homologue, can colocalize with the receptor cluster in the plasma membrane (Sourjik and Berg, 2000; Lipkow, 2006), and another possibility for HtrA accessibility would be if CheX were to interact with the flagellar motor of *B. burgdorferi*, where a three-dimensional model shows that it straddles the cell membrane and the periplasm (Charon *et al*., 2012).

HtrABb appears to be a functionally redundant effector with a potential role as a chaperone and a proteolytic role degrading outer membrane and signalling proteins. If HtrABb were to bind and protect all the co-immunoprecipitation partners that were identified in this study, we could suggest that it has the functions of a ‘traffic-cop’ in the movement of some proteins that are required for the adaptation of *B. burgdorferi* to its two hosts. We identified two substrates (BmpD and CheX) from a number of potential binding partners that were tested. The specificity of proteolysis indicates that HtrABb is not a promiscuous protease but rather one that can discriminate among its binding partners. Thus, we suggest that HtrABb can function as a chaperone for some of its binding partners and degrade others in a regulatory role. HtrABb is an important regulatory protease with predicted functions in degrading outer membrane lipoproteins and in regulating the chemotactic response of *B. burgdorferi*.

## Experimental procedures

### Generation of recombinant proteins

The open reading frame of *htrA*Bb, omitting the Signal Peptidase I recognition sequence, was amplified by PCR from *B. burgdorferi* strain B31A3 (Elias *et al*., 2002) using primers BB0104-5F and BB0104-2R (Table S1) and ligated into pre-digested expression plasmid pET28a (+) using restriction sites NdeI and XhoI, incorporating a 6× histidine tag at the NH_2_-terminus. Following confirmation of the construct by DNA sequencing, the resulting plasmid, p*htrA*Bb (Table S2), was transformed into *E. coli* BL21 Star(DE3)pLysS (Invitrogen, Grand Island, NY). Soluble protein was purified from the *E. coli* cytoplasm by affinity chromatography using a Histrap column (GE Healthcare Biosciences, Piscataway, NJ) as described previously (Coleman *et al*., 2009). Cleavage of the N-terminal His tag was carried out with the Thrombin Cleavage Capture Kit (Novagen, Billerica, MA) according to the manufacturer's instructions. Except where indicated, experiments were carried out using HtrABb with the His-tag attached. The final recombinant HtrABb buffer was PBS, pH 7.4, 5% glycerol.

Site-directed mutagenesis, using primers BB0104-S198AF and BB0104-S198AR (Table S1), was used to introduce a point mutation in the coding sequence for the catalytic domain of HtrABb, converting the catalytic serine198 to alanine (S198A) and has been described previously (Coleman *et al*., 2009). Soluble, purified HtrABbS198A protein was obtained as described for the wild-type protein.

The expression plasmid carrying the gene for basic membrane protein D (BmpD/BB0385) (gift of Dr Brian Stevenson, Department of Microbiology, Immunology, and Molecular Genetics, University of Kentucky College of Medicine, Lexington KY) was transformed into Rosetta (DE3)pLysS (Invitrogen). *E. coli* containing expression plasmids for CheX/BB0671 (M15/pQE-30) and FliL/BB0279 (Dh5a/pTRC-HIS) were the gift of Dr M. A. Motaleb, Department of Microbiology and Immunology, East Carolina University School of Medicine, Greenville, NC. BmpD (Verma *et al*., 2009), CheX (Motaleb *et al*., 2005) and FliL (Motaleb *et al*., 2011b) were expressed under conditions described previously and purified by use of MagneHis Protein Purification System (Promega, Madison, WI) (Verma *et al*., 2009). NapA (DPS)/BB0690 purified protein (Li *et al*., 2007) was the gift of Dr Erol Fikrig, Section of Infectious Diseases, Department of Internal Medicine, Yale University School of Medicine, New Haven, CT. Purified outer surface 22 kDa lipoprotein (lpLA7)/BB0365 (Pal *et al*., 2008) was the gift of Dr Utpal Pal, Department of Molecular Genetics and Microbiology, University of Maryland, College Park, MD. Purified OspA was the gift of Dr Justin Radolf, Department of Medicine, University of Connecticut Health Center, Farmington, CT. OspB was expressed and purified as described previously (Katona *et al*., 2000). Purity of recombinant protein was analysed by SDS-PAGE and Western blot as previously described (Coleman *et al*., 2009).

### Enzymatic assays

Proteolysis of fluorescein isothiocyanate-labelled casein was done as described previously (Twining, 1984; Coleman *et al*., 2009). Stock preparations of purified HtrABb and HtrABbS198A contained PBS and 5% glycerol. Final reaction conditions after addition of recombinant proteins were 1–125 μg ml^−1^ of recombinant protein, 100 μg of FITC-labelled casein, Type I (Sigma, St Louis, MO), 50 mM Tris pH 8.0, and 10 mM MgCl_2_ per reaction.

For experiments where casein degradation was shown by electrophoresis, HtrABb and HtrABbS198A (1 μg) were incubated at 37°C for 16 h in a 1.5 ml tube with 4 μg of casein (Sigma) in a final volume of 50 μl. SDS-PAGE sample buffer was added to 1× and the samples were boiled to terminate proteolysis. Following 12.5% SDS-PAGE, the gel was stained with Coomassie blue. Degradation of OspA, OspB, BmpD, CheX, FliL, NapA and P22/lpLA7 was done as described above, using 1 μg each of recombinant HtrABb, and 1–2 μg of recombinant target protein.

### Isolation of *B. burgdorferi* membrane vesicles

Mid log phase *B. burgdorferi* B31 (high passage or low passage strain A3) from a 500 ml culture were pelleted by centrifugation at 7000 *g* and resuspended in 50 ml of BSK II followed by incubation at 37°C for 2 h. Spirochaete cells were pelleted by centrifugation for 12 min at 7000 *g*, and the cell-free supernatant was filtered 2× using 0.22 μm Steriflip filters (Millipore). Vesicles were pelleted by ultracentrifugation for 1 h at 100 000 *g*. The filtered/centrifuged supernatant from this step was frozen at −80°C for later use (see *Detection of soluble HtrABb in* B. burgdorferi *conditioned medium*). The vesicles pellet was resuspended in 40% OptiPrep (Axis Shield, Oslo, Norway) diluted in 20 mM HEPES, pH 7.5. Two ml volumes of first 35%, then 30%, 25%, 20% and 15% OptiPrep were layered on top of the 40% OptiPrep/vesicles mixture to form a discontinuous step gradient. The gradient was centrifuged for 16 h at 100 000 *g* and 4°C. A white band containing the vesicles was visualized at the interface between the 20% and the 25% layers. Vesicles were collected and centrifuged for 1 h at 100 000 *g* followed by a wash step with 20 mM HEPES. Finally, the vesicles were resuspended in 1 ml of PBS and stored at 4°C until use.

### Preparation of total membranes and cytoplasm/periplasm fractions from *B. burgdorferi*

Late log phase *B. burgdorferi* (3 × 10^9^) were centrifuged at 7000 *g* for 15 min at 4°C. The cell pellet was washed 3× with Dulbecco's PBS, 5 mM MgCl_2_ (DPBS-Mg) and resuspended in 1 ml DPBS-Mg/1× protease inhibitor cocktail (EDTA-free) (Roche, Indianapolis, IN). The suspension was sonicated on ice for 2–3 min with a Microson Ultrasonic disruptor XL (Misonix Inc., Farmingdale, NY), at a power setting of 3–4. The sonicate was centrifuged at 7000 *g* to remove any remaining unbroken spirochaetes (darkfield microscopy revealed < 10 intact spirochaetes in the fifty 4× fields examined). The supernatant was transferred to a new tube and centrifuged at 100 000 *g* for 80 min at 4°C. The new supernatant containing the cytoplasm plus the periplasm was transferred to a new tube and the pellet, containing total membranes was resuspended in 1 ml DPBS-Mg. The samples were subsequently analysed by SDS-PAGE and Western blot.

### Detection of soluble HtrABb in *B. burgdorferi* conditioned medium

Cell-free, 2× filtered, centrifuged conditioned medium (see *Isolation of* B. burgdorferi *membrane vesicles*) was analysed directly for the presence of HtrABb by SDS-PAGE and Western blot. Uninoculated BSK II from the same batch used to make the conditioned medium was used as a control.

### Complementation of *E. coli* DegP high temperature growth-defective mutant JW0157-1 with HtrABb and *E. coli* DegP

Parental strain BW25113 and *degP* deletion mutant JW0157-1 (Δ*degP775::kan*), in which the *degP* open reading frame has been replaced by a *kan*-cassette, were obtained from the *E. coli* Genetic Stock Center (http://cgsc.biology.yale.edu). *E. coli* strain JW0157-1 grows normally at 37°C but does not grow at 43°C. Wild-type BW25113 grows to high density under both conditions. For complementation studies, the ORF for *htrA*Bb including the leader peptide was amplified by PCR using primers BB0104-17F and BB0104-18R and cloned into the EcoRI and KpnI sites of expression plasmid pBAD24 to form plasmid pBAD/*htrA*Bb-Bbls (Tables S1 and S2) (Guzman *et al*., 1995; Coleman *et al*., 2009). Growth-defective JW0157-1 was subsequently transformed with pBAD/*htrA*Bb-Bbls to form strain JW/pBAD/HtrA-Bbls.

To guard against the possibility that the *htrA*Bb leader sequence might not be recognized by the *E. coli* signal peptidase, *htrA*Bb was cloned into pBAD24 with the *E. coli* leader peptide substituting for its own. Fusion of the heterologous DNA was accomplished in three separate steps in which the open reading frame of *htrA*Bb was amplified by PCR from *B. burgdorferi* B31A3 DNA using 3′ primer BB0104-2Ra, which contained a KpnI restriction site, and 5′ primers Ecls-BB0104-1F, 2F and 3F that contained successive (25–27 bp) DNA sequences coding for the *E. coli* signal peptidase-1 recognition site (5′ primer Ecls-BB0104-3F contained an EcoRI restriction site, Table S1). Each PCR reaction used the previous product as template. The final insert consisted of DNA coding for the mature 1341 bp *htrA*Bb ORF immediately preceded by the 78 bp *E. coli* leader sequence flanked by the EcoRI and KpnI restriction sites. The construct was cloned into the region between the EcoRI and KpnI restriction sites of pBAD24 to form plasmid pBAD*htrA*Bb-Ecls, which was transformed into *E. coli degP* mutant JW0157-1 (Fig. S1) to form strain JW/pBAD/HtrA-Ecls. Arabinose-induced expression of HtrABb by the *E. coli* was assessed by SDS-PAGE/Western blot analysis. To demonstrate the efficacy of the complementation strategy, the *E. coli degP* (b0161) ORF (with the leader peptide) was cloned into pBAD24 using primers b0161-1F and b0161-2R (Table S1) to form plasmid pBAD/*degP*Ec. The Δ*deg*P strain JW0157-1 was transformed with pBAD/*degP*Ec. All constructs were verified by DNA sequencing. *E. coli* were cultured with shaking at 37°C and 43°C in Luria–Bertani medium (LB) containing different concentrations of arabinose to assess recovery of the wild-type growth phenotype.

### Co-immunoprecipitation of HtrABb and other *B. burgdorferi* proteins

To prepare cell-free lysate from *B. burgdorferi*, 400–500 ml of late log phase culture in BSK II medium was centrifuged (7000 *g*, 4°C, room temperature) and washed four times with 40 ml per wash of DPBS. The spirochaete pellet was suspended in 20 ml of 1× BugBuster Protein Extraction Reagent (EMD-Millipore, Billerica, MA) containing 20 μl of 100× Halt Protease Inhibitor Cocktail, EDTA-free (Thermo Scientific, Rockford, IL) and incubated for 20 min at room temperature with end-over-end rotation. The lysate was centrifuged for 15 min at 15 000 *g* and 4°C to pellet the debris. The supernatant was removed and centrifuged again for 5 min at 15 000 *g* and 4°C. The lysate was pre-cleared by an overnight incubation at 4°C with 30 mg ml^−1^ Dynabeads Protein G prior to use (Immunoprecipitation Kit-Protein G, Invitrogen). Rabbit anti-HtrABb Ig (10 μg ml^−1^), prepared from serum by use of the Melon Gel IgG Spin Purification Kit (Thermo Scientific), was incubated with 30 mg of Dynabeads Protein G for 10 min and the beads were washed one time with Kit Ab Binding and Wash Buffer. The Dynabeads-Ab complex was then incubated end-over-end at room temperature for 10 min with 400–600 μl of *B. burgdorferi* lysate followed by four 200 μl washes with Kit Wash Buffer. The Dynabeads were resuspended in 30 μl of 1× SDS-PAGE sample buffer and boiled for six min. Following 12.5% SDS-PAGE, the gels were stained with 0.1% Coomassie blue in 50% methanol and 10% acetic acid. To control for non-specific binding, co-immunoprecipitation was also done separately with pre-immune serum IgG from the same rabbit used for immunization. Separate co-immunoprecipitations were done with no lysate or no IgG.

### Size exclusion chromatography

A HiLoad 16/60 Superdex 200 prep grade column (GE Healthcare Life Sciences) equilibrated in 50 mM Tris (pH 7.5) containing 240 mM NaCl and 5 mM EDTA at 20°C was used for the gel filtration chromatography experiments. A gel filtration calibration kit (high molecular weight; GE Healthcare Life Sciences) was used to calibrate the column. Experimental protein samples (500 μl) at concentrations between 0.1 and 0.5 mg ml^−1^ were applied to the column depending on the experiment. The fractions were collected in 2.5 ml volumes and analysed by SDS-PAGE.
