# Supplementary material for: The HtrA protease of Borrelia burgdorferi degrades outer membrane protein BmpD and chemotaxis phosphatase CheX
Source: Mol Microbiol. 2013 Apr 9;88(3):619–33. doi: 10.1111/mmi.12213 (PMC3641820; doi:10.1111/mmi.12213)
Supplement: Supplementary file 1 [file mmi0088-0619-SD1.pdf]

Table S1. Primers used in this study[a]

Primer	RS[b]	Primer Sequence (5'-3')	Purpose
BB0104-2R	XhoI	TAATCCTCGAGCTAAAATGTAATTTTAAAAGAA TCGTTT	Cloning of HtrABb ORF into pET28a
BB0104-5F	NdeI	GGAATTCCATATGTCTAATAGAAGCAACATTGTTTTGC	Cloning of HtrABb ORF into pET28a
T7PF	-	TAATACGACTCACTATAGGG	Sequencing of pET28a/HtrABb
T7tR	-	GCTAGTTATTGCTCAGCGG	Sequencing of pET28a/HtrABb
BB0104-7F	-	GTGGTAGATAAGGCAACTGAAC	Sequencing of pET28a/HtrABb
BB0104-8F	-	CGCTAAAGACAAGAGATTCTGAGG	Sequencing of pET28a/HtrABb
BB0104-9R	-	GTTCAGTTGCCTTATCTACCAC	Sequencing of pET28a/HtrABb
BB0104-10R	-	CAGAATCTCTTGTCTTTAGCGG	Sequencing of pET28a/HtrABb
pBADP	-	TTTATCGCAACTCTCTACTGT	Sequencing of pET28a/HtrABb
pBAD24-1R	-	TCCGCCAAAACAGCCAAGC	Sequencing of pET28a/HtrABb
BB0104-S198AF	-	CAACAGGGGTAATgCAGGTGGTCCTCTTGTAA	HtrABb site-directed mutagenesis
BB0104-S198AR	-	GGACCACCTGcATTACCCCTGTTGATTGC	HtrABb site-directed mutagenesis
BB0104-17F	EcoRI	TAATGAATTCACCGTGGAAAAAAAGTTTTTTTCTG	Cloning of HtrABb-BbIs[c] into pBAD24
BB0104-18R	KpnI	TAATGGTACCCTAAAATGTAATTTTAAAAGAATCG	Cloning of HtrABb-BbIs[c] into pBAD24
EcIs-BB0104-1F	-	GCGTTATCTCCGCTCTCTGCAACGGCGTCTAATA<br>GAAGCAACATTGTTTTTG	Cloning of HtrABb-EcIs[d] into pBAD24
EcIs-BB0104-2F	-	GTGCACTGGCTCTGAGTTTAGGTTTGGCGTTATC<br>TCCGCTCTCTGC	Cloning of HtrABb-EcIs[d] into pBAD24
EcIs-BB0104-3F	EcoRI	TAATGAATTCACCATGAAAAAAACCACATTAGCAC<br>TGAGTGCACTGGCTCTGAGTTTAGG	Cloning of HtrABb-EcIs[d] into pBAD24
BB0104-2Ra	KpnI	GCCGGGTACCCTAAAATGTAATTTTAAAAGAATCG<br>TTTCCTCTCAAAATTTT	Cloning of HtrABb-EcIs[d] into pBAD24
b0161-1F	EcoRI	TAATGAATTCACCATGAAAAAAACCACATTAGC	Cloning of Ec degP into pBAD24
b0161-2R	KpnI	GCCGGGTACCTTACTGCATTAACAGGTAGATG	Cloning of Ec degP into pBAD24
b0161-3F	-	CCGATAAAGGCTATGTCGTC	Sequencing of Ec degP[e]
b0161-4F	-	TGGCGAAAGCGATGAAAG	Sequencing of Ec degP[e]
b0161-5R	-	TGACGACATAGCCTTTATCG	Sequencing of Ec degP[e]
b0161-6R	-	TTTCGCCAGTTCGGAGTTC	Sequencing of Ec degP[e]

[a]All PCR primers shown here with the exception of T7PF and T7tR were designed for this study
[b]Restriction site underlined
[c]HtrABb cloned with own leader sequence
[d]HtrABb cloned with *E. coli* leader sequence instead of own leader sequence
[e]*E. coli* degP cloned with own leader sequence

Table S2. Bacterial strains used and plasmids generated in this study

	Strain or plasmid	Genotype or description	Reference or source
B. burgdorferi	B31A3	cp9-, wild-type	Elias *et al.*, 2002
E. coli	BW25113	Parent, *Δ(araD-araB)567, ΔlacZ4787(::rrnB-3), λ–, rph-1, Δ(rhaD-rhaB)568, hsdR514*	Keio Col., *E. coli* Genetic Stock Center, Yale Univ., New Haven, CT
	JW0157-1	*Δ(araD-araB)567, ΔdegP775::kan, ΔlacZ4787 (::rrnB-3, λ–, rph-1, Δ(rhaD–rhaB)568, hsdR514*	Keio Collection, *E. coli* Genetic Stock Center
	JW/pBAD	JW0157-1 containing pBAD24	This study
	JW/pBAD/*HtrA*Bb-Bbls	JW0157-1 containing pBAD/*htrABb*-Bbls	This study
	JW/pBAD/*HtrA*Bb-Ecls	JW0157-1 containing pBAD/*htrABb*-Ecls	This study
	JW/pBAD/*degP*Ec	JW0157-1 containing pBAD/*degP*Ec	This study
	BL21 Star(DE3)pLysS	Expression host, F– *omp*T *hsd*SB(rB–, mB–) *gal dcm rne*131 (DE3) pLysS (CamR)	Invitrogen, Carlsbad, CA
	Rosetta(DE3)pLysS	Expression host, F– *omp*T *hsd*SB(rB–, mB–) gal*dcm* (DE3) pRARE (CamR)	Novagen, Gibbstown, NJ
	DH5α/pTRC-HIS	Expression clone (FliL)	Motaleb *et al.*, 2011a
	M15/pQE-30	Expression clone (CheX)	Motaleb *et al.*, 2005
	Rosetta(DE3)pLysS/*pbmpD*	Expression clone (BmpD)	This study
Plasmids	pET28a	Expression plasmid	Novagen
	pBAD24	Expression plasmid	Guzman *et al.*, 1995
	p*htrABb*	pET28a containing WT *htrABb*	This study
	p*htrABb*S198A	pET28a containing *htrABb*S198A	This study
	pBAD/*htrABb*-Bbls	pBAD24 containing *htrABb* and Bb leader sequence	This study
	pBAD/*htrABb*-Ecls	pBAD24 containing *htrABb* and Ec leader sequence	This study
	pBAD/*degP*Ec	pBAD24 containing *degPEc* and Ec leader sequence	This study
	p*BmpD*	Expression plasmid containing *bmpD*	Verma *et al.*, 2009

Table S3. Predictive Algorithms used for CheX

Server	Web address	Prediction
HMMTOP	http://www.enzim.hu/hmmtop/index.php	Transmembrane α-helix for aa residues 45 - 59
“DAS”-Transmembrane Prediction	http://www.sbc.su.se/~miklos/DAS/	Transmembrane α-helix for aa residues 44 - 56 with a 1.7 value near strict cutoff of 2.
TMpred	http://www.ch.embnet.org/software/TMPRED_form.html	Strongly preferred model of an inside to outside transmembrane α-helix for aa residues 43 -59 with a value of 897, where values >500 are considered significant. Model assumes an outside loop beyond aa residue 60.
Mobyle	http://mobyle.pasteur.fr/cgi-bin/portal.py	Putative transmembrane α-helix for aa residues 41 - 61 (score 0.946). External loop from aa residues 62 - 161 was predicted for CheX.
Peptide Cutter	http://web.expasy.org/peptide_cutter/	13 Trypsin cleavage sites for CheX with 8 of these located in the external loop (aa residues 72,87,112,130,134,149,158,160)
SignalP 4.0	http://www.cbs.dtu.dk/services/SignalP/	CheX does not have a signal peptide sequence

Figure S1.

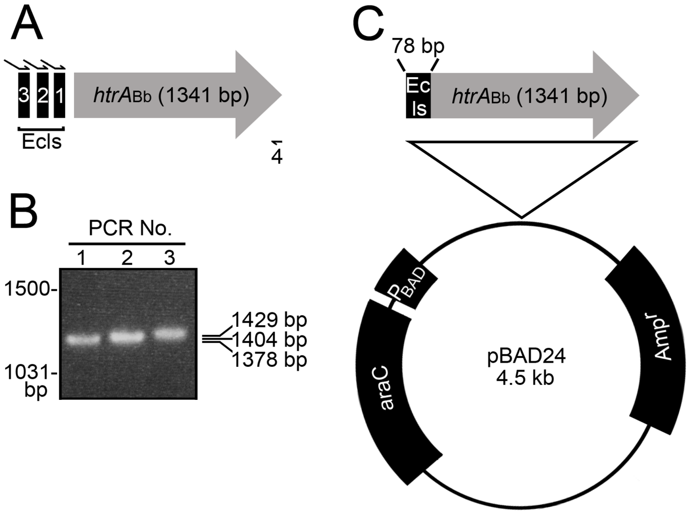
A
3
2
1
htrABb (1341 bp)
Ecls
4
B
PCR No.
1
2
3
1500-
1031-
bp
1429 bp
1404 bp
1378 bp
C
78 bp
Ec
ls
htrABb (1341 bp)
PBAD
araC
pBAD24
4.5 kb
Ampr
